# Protocol for a Study Investigating Context-Specific Sedentary Behaviors and Cardiometabolic Health in College-Based Young Adults (CONTEXT-SB)

**DOI:** 10.21203/rs.3.rs-4470004/v1

**Published:** 2024-06-11

**Authors:** Jake C. Diana, Aiden J. Chauntry, Emma Cowley, Craig Paterson, Jeb Struder, Patricia Pagan-Lasalle, Michelle L. Meyer, Feng-Chang Lin, Justin B. Moore, Erik D. Hanson, Lee Stoner

**Affiliations:** University of North Carolina at Chapel Hill; University of North Carolina at Chapel Hill; Liverpool John Moores University; University of Bristol; University of North Carolina at Chapel Hill; University of North Carolina at Chapel Hill; University of North Carolina at Chapel Hill; University of North Carolina at Chapel Hill; Wake Forest University; University of North Carolina at Chapel Hill; University of North Carolina at Chapel Hill

**Keywords:** Socioecological Model, Ecological Momentary Assessment, Cardiometabolic Disease, Accelerometry, Pulse Wave Velocity, Television Viewing

## Abstract

**Background:**

Sedentary behavior (SB) is detrimental to cardiometabolic disease (CMD) risk, which can begin in young adulthood. To devise effective SB-CMD interventions in young adults, it is important to understand which context-specific sedentary behaviors (CS-SB) are most detrimental for CMD risk, the lifestyle behaviors that co-exist with CS-SBs, and the socioecological predictors of CS-SB.

**Methods:**

This longitudinal observational study will recruit 500 college-aged (18–24 years) individuals. Two laboratory visits will occur, spaced 12 months apart, where a composite CMD risk score (e.g., arterial stiffness, metabolic and inflammatory biomarkers, heart rate variability, and body composition) will be calculated, and questionnaires to measure lifestyle behaviors and different levels of the socioecological model will be administered. After each visit, total SB (activPAL) and CS-SB (television, transportation, academic/ occupational, leisure computer, “other”; ecological momentary assessment) will be measured across seven days.

**Discussion:**

It is hypothesized that certain CS-SB will show stronger associations with CMD risk, compared to T-SB, even after accounting for coexisting lifestyle behaviors. It is expected that a range of intra-individual, inter-individual, and physical environment socioecological factors will predict CS-SB. The findings from this study will support the development of an evidence-based, multi-level intervention to target SB reduction and mitigate CMD risk in CBYA.

## 1. Introduction

Cardiometabolic disease (CMD) is a critical health burden in young adults, with approximately 10–20% of this population exhibiting advanced but asymptomatic atherosclerotic lesions^1–3^. CMD trajectories in this population are also worsening, with type 2 diabetes prevalence increasing from 0.9% (1971–75) to 3.2% (2009–2012) among adults aged 20–44 years, exceeding the trajectory in those older than 45 years^4^. In the United States, two-thirds of young adults attend college^5,6^, where they often adopt poor lifestyle behaviors^7,8^ that track into older age and contribute to CMD development^9,10^. Consequently, young adulthood is an important, yet under-researched period to implement lifestyle behavior interventions that aid the primary prevention of CMD.

SB is a modifiable lifestyle factor defined as low intensity (< 1.5 metabolic equivalents [METs]) seated, reclined or supine behaviors^11^. SB is biologically distinct from physical inactivity (defined as not meeting physical activity guidelines), such that individuals can be both highly sedentary and highly physically active^12^. In college-based young adults (CBYA), SB has been researched far less than other lifestyle behaviors^13,14^. This is despite SB being the dominant waking behavior in this population, with accelerometry-derived estimates of 10–11 hours/day^15,16^. Worryingly, a moderate-to-strong association exists between total SB and CMD risk^16^, even in those meeting moderate-to-vigorous physical activity (MVPA) guidelines^17,18^. Consequently, public health agencies such as the World Health Organization have called on the research community to better understand how to implement SB-reduction interventions^14,19^.

To develop effective SB-CMD interventions, it is necessary to fully elucidate the relationship between SB and CMD, but this relationship is poorly understood in CBYA. This is likely due to the multidimensional complexity of SB which occurs across a wide spectrum of contexts, including television viewing, occupational sitting, seated transport, and leisure-time computer use. Cross-sectional research in general adult populations using retrospective self-report measures indicates that television viewing is more strongly associated with CMD risk, relative to other CS-SBs^19^. The reasons for this are not well understood, but may include simultaneous engagement with unhealthy behaviors, such as the (over-)consumption of processed foods^20^. However, whether total or CS-SB is more strongly associated with CMD risk, and how coexisting lifestyle behaviors influence this association, is not known in CBYA.

We also have scant understanding of the variables that predict CS-SB in CBYA, but this information is critical for the development of successful SB interventions. The socioecological model (SEM) has been used to increase MVPA levels^21^, and highlights that SB interventions are unlikely to be successful unless multiple levels of SB are considered, including intrapersonal (e.g., awareness of time spent engaging in SB), interpersonal (e.g., friends involvement with SB), and physical environmental (e.g., living environment) factors^21–23^. One longitudinal study found that social-cognitive variables like attitudes, self-efficacy, and social norms were the strongest CS-SB predictors, while physical environment factors showed limited effects^24^. However, this line of research has solely been conducted in general adult populations, and has predominantly focused on non-modifiable socio-demographic variables^23,25^, which ignores the full spectrum of SEM factors^26^.

In summary, CMD is a growing concern in CBYA, and therefore it is critical to develop effective interventions and public health recommendations for this population. However, in CBYA we do not yet fully understand the link between CS-SB and CMD risk, the influence of behaviors that co-exist with CSSB on CMD risk, and the socioecological predictors of CS-SB. Without this information, it is not possible to devise highly effective SB interventions that are capable of mitigating CMD risk. Therefore, the aims of the Cardiometabolic Outcome Negation Through Early-adulthood ConteXT-specific Sedentary Behavior reduction (CONTEXT-SB) study are:

### Aim 1

To identify whether total T-SB or CS-SB is more strongly associated with CMD risk in CBYA.

### Aim 2

To determine whether the Aim 1 CMD risk is directly explained by SB or mediated by co-occurring lifestyle behaviors (e.g., diet, physical activity) that cluster with SB.

### Aim 3

To investigate which SEM factors (intra-individual, inter-individual, physical environment) are associated with SB.

The overall deliverable (underpinned by the three aims above) of CONTEXT-SB is to develop an evidence-based, multi-level intervention that targets SB reduction in CBYA.

## 2. Methods

### 2.1 Study Design & Overview

**Figure 1:**
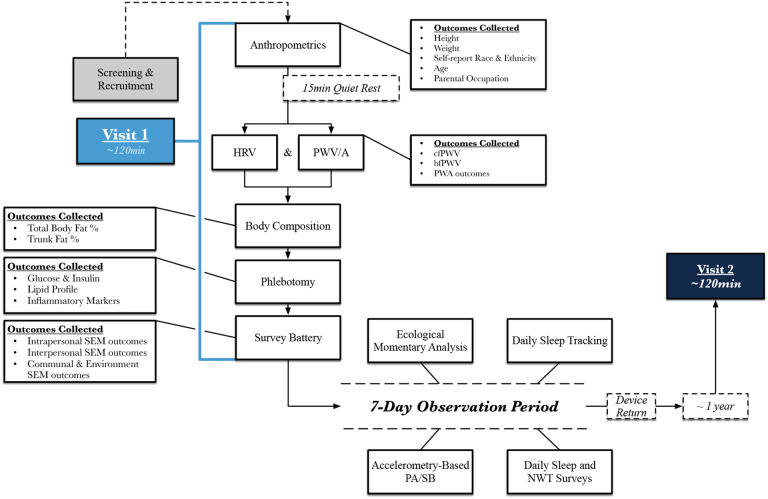
Visualization of the CONTEXT-SB study protocol. After enrolling in the study, participants are scheduled for their Visit 1 assessments at the Cardiometabolic Lab, where cardiometabolic disease risk is established and movement behavior devices are deployed. The Visit 2 protocol is identical to Visit 1. *Abbreviations: bfPWV, Brachial-femoral pulse wave velocity; cfPWV, Carotid-femoral pulse wave velocity; HRV, Heart rate variability; min, Minutes; NWT, Non-wear time; PA, physical activity; PWA, Pulse wave analysis; PWV, Pulse wave velocity; SB, Sedentary behaviors.*

CONTEXT-SB is a longitudinal observational study conducted within the Cardiometabolic Laboratory (CML) at the University of North Carolina at Chapel Hill (UNC-CH). There are two identical laboratory visits, each lasting ~ 120 minutes, separated 12 months apart (Fig. 1). After the completion of each laboratory visit, a seven-day movement behavior assessment period occurs, which includes measuring CS-SB and T-SB. The study has received full ethical approval (IRB #22–0819), and participants provide informed consent at the start of the first laboratory visit.

### 2.2 Inclusion Criteria

We aim to recruit 500 CBYA from UNC-CH. Inclusion criteria are: (i) college students aged 18–24 years, (ii) available for follow-up testing 12 months after the first lab visit, and (iii) plan to be on campus at least one month prior to the follow up laboratory visit. An inclusive recruitment strategy has been adopted in an attempt to better reflect the diverse health and behavioral characteristics of CBYA. Before each lab visit, the following pre-assessment guidelines are followed: fasted for 12 hours; no alcohol consumption for 24 hours; no consumption of supplements for 24 hours; no strenuous/vigorous exercise for 24 hours.

### 2.3 Recruitment, Screening & Retention

To ensure the sample is demographically (e.g., age, sex, race) representative of the UNC student body, a quota sample approach is used. Our primary recruitment strategies are classroom visits, flyers, mass emails, and a UNC-CH participant recruitment website. To boost recruitment and retention, flexible scheduling (weekends) and automated follow-up reminder messages via the Calendly software^27^ are utilized. Participants receive a $50 gift card for each study visit and are entered into a random prize draw for an Apple Watch. It is conservatively estimated that 80% of participants (n = 400) will complete both study visits.

### 2.4 Assessment Visits

Upon arrival at the CML, pre-assessment guideline adherence is confirmed. Acquisition of consent (DocuSign) and the completion of each aspect of the study protocol is recorded via an online electronic database (REDCap)^28,29^. A full visualization of the study protocol is provided in Fig. 1.

### 2.5 Primary Outcome – Composite Cardiometabolic Disease Risk Score

The primary outcome measure for CONTEXT-SB is a composite CMD risk score, which is comprised of 14 novel and traditional markers of cardiometabolic health (see Table 1).

#### Cardiovascular outcomes

The VICORDER^®^ system (SMT Medical Technology GmbH, Wuerzburg, Germany) measures carotidfemoral pulse wave velocity [cfPWV] and brachial-femoral pulse wave velocity [bfPWV]), which are markers of arterial stiffness (AS). There are 15 minutes of quiet rest before the first measurement, and all measures are taken in triplicate with one minute of quiet rest in between; the closest two being averaged for later analysis. In accordance with the manufacturer’s guidance, participants are positioned in a semi-recumbent posture at an incline of 25°. This positioning helps to minimize potential interference from the jugular vein in the detection of the foot of the carotid upstroke. Briefly, the device calculates pulse transit time via oscillometry at two arterial sites, and manufacturer guidelines were followed for capturing the straight-line distances between measurement sites. For measures of cfPWV, the straight-line distance from the carotid artery to the femoral artery is multiplied by a factor of 0.8, to more accurately reflect the actual physiological path distance^30^. Additionally, the VICORDER^®^ system calculates oscillometric resting blood pressure and additional outcomes from pulse wave analysis (PWA), including augmentation index, central systolic blood pressure, and mean arterial pressure.

The MindWare Mobile system (MindWare Technologies, Ltd., Westerville, OH) receives signals from 3-lead electrocardiography (ECG) and 4-lead impedance cardiography (ICG), which are processed continuously using BioLab software. Data are collected for the last 5 minutes of the 15-minute resting period in a semi-recumbent position. Guidelines for quality control are followed, including cleaning electrode sites with 70% ethanol prior to electrode placement^31,32^. A visual assessment of high-quality signal is confirmed before data collection is initiated. The primary heart rate variability (HRV) outcome is the root mean square of the successive difference (RMSSD). Secondary outcomes from HRV may include non-linear HRV metrics (e.g., entropy) and frequency-domain parameters such very-low frequency (0.00–0.04 Hz), low frequency (0.04–0.15 Hz), and high frequency (0.15–0.40 Hz) paramaters^33^. Outcomes from ICG are secondary measures and include stroke volume and cardiac output.

#### Body Composition Outcomes

Full body dual-energy x-ray absorptiometry scans (DXA; Hologic, Horizon; Bedford, MA) are performed to assess total body fat and trunk fat percentage. Participants are instructed to wear clothing without metal and to remove accessories prior to the scan. Participants are positioned supine according to manufacturer guidelines with arms placed to the side, palms pronated, and legs internally rotated such that the toes are touching. If a participant’s height exceeds the examination area, the lower limbs are prioritized, as head body composition is estimated. Height and body mass are also collected at the initiation of each study visit using a standard stadiometer and scales.

#### Metabolic and Inflammatory Outcomes

Two 10mL whole blood samples were collected within serum and ethylenediamine tetra-acetic acid (EDTA [K2]) tubes (BD Vacutainers, Becton, Dickinson and Company, Franklin Lakes, NJ, USA). Immediately post-collection, 1.5μL of EDTA whole blood is used for assessing glycated hemoglobin (HbA1c) via a multi-assay analysis platform (AFINION^™^ 2 Analyzer, Abbott Diagnostics Technologies AS, Oslo, Norway), while the serum sample is allowed to clot at room temperature for one hour. Both samples are centrifuged at room temperature for 15 minutes at 1200 × g. A 40 μL sample of the serum supernatant is used to generate a hematological metabolic profile (e.g., triglycerides, high-density lipoprotein cholesterol, low-density lipoprotein cholesterol, fasting blood glucose) via reflectance photometry (Cholestech LDX^™^ Analyzer, Abbott Diagnostics Technologies AS, Oslo, Norway). Remaining supernatants are aliquoted and stored at – 80° C until future analysis. Planned outcomes include insulin and C-reactive protein, which will be assessed via ELISA according to manufacturer instructions.

### 2.6 Aim 1 Exposure Variables

The aim 1 exposure variables are T-SB and five CS-SBs.

#### Context-Specific Sedentary Behavior

**Figure 2:**
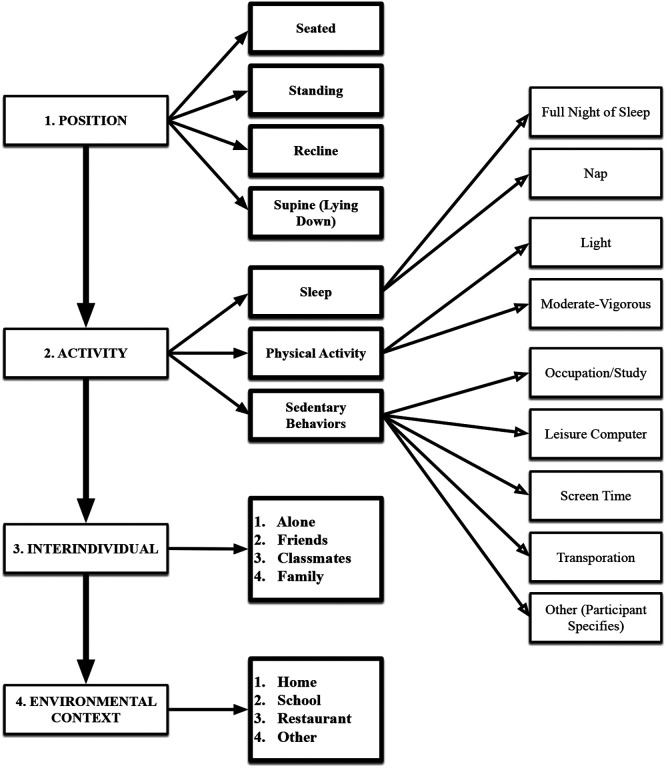
Ecological Momentary Assessment (EMA) sequence. Participants are asked to reflect on the context-specific sedentary behaviors they were engaging in at the moment they received the survey.

The Smartphone Ecological Momentary Assessment (SEMA^3^; Melbourne eResearch Group, Melbourne, Australia) application is installed on each participant’s personal mobile device^34^. Participants are promoted to complete nine daily surveys, with one survey programmed to be distributed during each of these blocks: 00:00–02:15, 02:45 − 05:00, 05:30 − 07:45, 08:15 − 10:15, 10:45 − 12:45, 13:15–15:15, 15:45 − 18:00, 18:30 − 20:45, 21:15–23:30. This approach was selected account for the varied sleeping patterns of CBYA^35^. The surveys measure a range of intra-individual, inter-individual and environmental factors, including CB-SB (occupation/study; leisure computer; screen time; transportation; other - see Fig. 2). Participants are instructed to reflect on their behavior immediately prior to receiving the survey, and to complete the surveys as quickly as possible, as each survey becomes unavailable 30 minutes post-deployment. Participants are alerted via the SEMA3 application if they fall below a set compliance threshold of 60%.

#### Total Sedentary Behavior

The activPAL (PAL Technologies Ltd.; Glasgow, UK) is a gold-standard inclinometer which records data at 20 Hz (2 seconds) and is affixed on the anterior portion of the right thigh (midpoint between the anterior superior iliac spine bony landmark and knee bone) using a nitrile/latex sleeve with waterproof Tegaderm dressing. Participants are instructed to remove the device only for high-impact contact sports, or during swimming in deep environments where it may be lost. The activPAL captures sedentary behavior (T-SB) data for seven full days (24h/day), beginning at 11:59pm the day of each study visit, using established methods^36,37^. The PALanalysis software will be used to calculate T-SB and other SB outcomes using the CREA algorithm^36–38^. Following National Health and Nutrition Examination Survey protocols^39,40^, a valid day of activPAL monitoring is defined as at least 16 hours of wear time, with 3 valid weekdays and 1 valid weekend day being required for data inclusion.

An automated survey is sent to participants via REDCap each morning of the seven-day movement behavior assessment period. Participants report their sleep, wake, and nap times. This information is collected to cross-validate sleep data from the objective sleep device (see below) and activPAL. Participants also report any time periods in which the activPAL was removed, to quality check the non-wear times estimated by the activPAL.

For a small subset (n = 20) at the start of the study, the MOX 1 accelerometer (Maastricht Instruments BV; Maastricht, NL) was used. However, a lack of functionality to detect non-wear and sleep periods was found to be prohibitive for continued use in the study. To ensure within-subject data comparability, the same accelerometer will be used for both visit 1 and visit 2.

#### Combination of EMA and activPAL Data

Using a custom R script, the activPAL epoch-level data will be aggregated into 30-minute segments (e.g., 01:00–01:30, 10:30 − 11:00, 16:00–16:30). Similarly, the timestamped ecological momentary assessment (EMA) survey responses will be merged with the activPAL data, assigned to the appropriate 30-minute time block (e.g., a survey completed at 14:15 will be assigned to the 14:00–14:30 block). These data will then be combined to calculate time spent (per week) in each CS-SB. To offer an example, a participant may engage in 60 hours of activPAL-measured T-SB throughout the seven-day observation period. The EMA data shows that a participant responded to 52 out of the 63 possible surveys (9 surveys per 7 days of observation). Of these 52 responses, 40 were reported to be SB, and 30 of these 40 SB responses were identified to be in the occupation/study domain. Thus, time spent in occupational/study-based SB is calculated as 45 hours across the week or 75% (30/40*100).

### 2.7 Aim 2 Exposure Variables

Below (and see Table 2) are the 11 lifestyle behavior exposure variables for Aim 2, which may partially explain associations between T/CS-SB and CMD risk.

#### Diet and Substance Use

Dietary patterns will be derived from the Diet Health Questionnaire III (DHQIII; 154 items)^41^. Specifically, we expect principal component analysis to generate three dietary patterns: processed foods, fruit and vegetable consumption, and breakfast foods.

The Alcohol, Smoking, and Substance Involvement Screening Test (12 items, ICC = .90 to .97)^42^ will measure specific involvement scores for 12 different substances (tobacco, alcohol, cannabis, cocaine, prescription stimulants, methamphetamines, inhalants, sedatives or sleeping pills, hallucinogens, heroin, prescription opioids, and other substances). For the primary analysis, we will derive three variables which are considered most specific to our CBYA population: a score for tobacco and alcohol, and a global risk score which sums together all drug classes.

#### Sleep

Objective sleep data will be captured using the SleepScore Max device (Sleep Solutions LLC, Carlsbad, CA), linked to the SleepScore application downloaded on each participant’s personal mobile device. In line with manufacturer guidelines, participants are instructed to rest the device at chest level on a surface next to their bed for all sleeping and napping periods across the 7-day observation period. The device uses sonar technology to non-invasively track sleep without coming into contact with the participant and potentially interfering with natural sleeping patterns. The SleepScore Max device has been validated against polysomnography (sensitivity = 0.94, accuracy = 0.88)^43^. Participants are instructed to activate the SleepScore Max device immediately before they begin sleeping and to end the sleep tracking immediately when they awake from each bout of sleep. Both summarized and daily epoch data are exported for later analysis.

In addition, the 5-item Reduced Morning-Eveningness Questionnaire^44^ (rMEQ) is administered to examine the sleep chronotype of each participant (e.g., morning-oriented or evening-oriented).

The 3 primary sleep exposure variables will be sleep duration, sleep quality/efficiency (SleepScore Max) and sleep chronotype (Reduced Morning-Eveningness Questionnaire).

#### Physical Activity

Light physical activity (LPA) and moderate-to-vigorous physical activity (MPVA) will be derived from the raw acceleration data acquired from the activPAL and MOX1 accelerometers (see above). This data will be derived in R using the GGIR package^45,46^. Established cut points for raw acceleration data from the thigh will be applied in analyses^47^. Validation criteria for valid days is described above in the activPAL SB section.

### 2.8 Aim 3 Exposure Variables

Sixteen socioecological model variables will form our Aim 3 exposure variables (see Table 3). These were selected with consideration to theoretical models, previous population use, and brevity (88 total items).

#### Intra-individual

Five intra-individual variables will be measured (see Table 3). Self-Efficacy will be assessed using a modified version of the Physical Activity Self-Efficacy scale (PASE)^48^. Self-Regulation, Outcome Expectations, and Personal Barriers will be evaluated through a modified Cognitive Behavioral Physical Activity Questionnaire (CBPAQ)^49^. Additionally, psychological stress will be measured using the Perceived Stress Scale^50^ and the Personal Burnout Scale^51^.

#### Inter-individual

Two inter-individual variables will be measured: Social Norms and Social Support. Social Norms will be evaluated by adapting the scale used by Ball *et al*^52^. Participants will respond to the following statements using a 5-point Likert scale: (i) “I often see other people purposefully interrupting their sedentary behavior (SB); (ii) “Lots of people I know purposefully interrupt their SB”; and (iii) “Lots of people I know regularly engage in SB for long periods without interruption.” Social Support will be measured by adapting the scale utilized by Sallis *et al*^53^. Using a 5-point Likert scale, respondents will rate the frequency with which friends or colleagues, during the past year: (i) purposefully interrupted my SB; (ii) encouraged me to reduce SB; and (iii) discouraged me from excessive SB.

#### Physical environment

Four physical environment variables will assess participants’ perceptions of the college environment and their current residence, regarding: (i) Functionality, (ii) Safety, (iii) Aesthetics, and (iv) Destinations. These variables will be evaluated using the Physical Environmental Neighborhood Factors (PENF) scale^54^.

### 2.9 Covariates

Additional standard surveys to collect demographic (e.g., age, sex, race, ethnicity) information will also be administered, as will the ISCO-88 to classify parental occupation (e.g., manager, professional, service and sales worker) based on the primary occupation of the adult in the family household who earns the most income (i.e., primary breadwinner)^55^.

### 2.10 Statistical Methods and Sample Size Consideration

The primary outcome is a composite CMD risk score derived using factor analysis from the outcomes shown in Table 1^56^. The number of factors will be determined using parallel analysis and cross-validation. The factors will then be subject to orthogonal ‘varimax’ rotation. If varimax rotation fails to produce interpretable factors, non-orthogonal rotations will be implemented. We will use an a priori loading greater than 0.40 to interpret the factor pattern. We will derive a CMD risk score from this factor structure by summing the individual factor scores^56^.

#### 2.10.1 Aim 1 Analysis

Associations between CMD risk with T-SB and each of the five CS-SB (see Fig. 2) will be estimated using linear mixed-effects models. An exchangeable working covariance structure will account for repeated measures within the same individual over time.

#### 2.10.2 Aim 2 Analysis

A two-stage approach will be used^57^. First, we will conduct agglomerative hierarchical cluster analyses based on the Ward method with squared Euclidean distance to identify the potential number of clusters among participants and calculate the initial centroid values for each cluster. Second, we will perform a k-means cluster analysis using the number of clusters identified in stage one to form the final clustering result. To examine the stability of the final clustering, we will randomly divide the total sample into two halves, and the described process will be repeated. Cohen’s k coefficients will be calculated to measure the degree of agreement between the classification of participants using the total sample and each half-subsample.

Longitudinal mediation effects will be examined using the two-step longitudinal parallel process (LPP)^58^. In the first step, the intercept and slope of each longitudinal variable will be estimated separately for each individual using multilevel modeling. This approach will account for any missing longitudinal data. In the second step, separate structural equation models will test for the longitudinal mediation effects of T/CS-SB on CMD risk for the lifestyle behavior(s) (Table 2) in each cluster. Additionally, if the data are non-normally distributed, bootstrap resampling will be implemented to obtain more stable and valid estimates of standard errors^59^. The final model will be adjusted for age, sex, race, and parental occupation.

#### 2.10.3 Aim 3 Analysis

Associations between T/CS-SB and each SEM variable (Table 3) will be evaluated using separate linear mixed-effects models. An exchangeable working covariance structure will account for repeated measures within the same individual over time. The SEM variables will be specified separately, then collectively, using a stepwise model. The final model will be adjusted for age, sex, race, and parental occupation. While not a primary aim, in a secondary analysis we will additionally test sex and race as potential moderators and conduct stratified analysis if appropriate.

#### 2.10.4 Sample Size Justification

The recruitment target for CONTEXT-SB is N = 500 individuals, which we anticipate will result in a total of 900 observations - assuming two observations per participant with 80% retention for visit 2.

##### Aim 1 -

Two different approaches are commonly used to estimate sample size in factor analysis: (i) a minimum participant-to-item ratio ranging from 5:1 to 10:1^60,61^; or (ii) a minimum number of participants ranging from 50 to 400^62–64^. We opted to satisfy the very good to excellent criteria suggested by Comrey and Lee, where: 50 – very poor; 100 – poor; 200 – fair; 300 – good; 500 – very good; >1000 – excellent^65,66^. The optimal T/CS-SB measure will be determined using mixed model linear regression. The test of ρ2=0 (α = 0.05) for five normally distributed covariates (SB plus five adjustment covariates) will have 80% power to detect a ρ2 of 0.015.

##### Aim 2 -

There are no generally accepted guidelines to estimate sample size for cluster analysis. At a minimum, 10 times the number of clustering variables has been recommended. With two observations and assuming a conservative intra-class correlation (ICC) of 0.4, a sample size of 202 is required to detect a medium direct and a small indirect effect (mediation) with 80% power. The sample size increases to 341 when both the direct and indirect effects are small.

##### Aim 3 -

The mixed model multiple linear regression test of ρ2=0 (α = 0.05) for 16 normally distributed covariates will have 80% power to detect a ρ2 of 0.021.

## 3. Results and Discussion

### 3.1 Expected Results

#### Aim 1

Based on emerging literature, it is anticipated that time spent in certain CS-SB will exhibit stronger associations with CMD risk scores relative to T-SB^67,68^. Specifically, we hypothesize that screen time will be most deleterious, based on the stronger associations seen between TV viewing (relative to other CS-SB) and CMD risk in general adult populations^69,70^. A recent meta-analysis in young adults revealed that incidence of hypertension and dyslipidemia significantly increased with each additional hour of cumulative TV watching^71^. However, it is important to note these studies relied on retrospective self-report, which is prone to recall bias^72^, highlighting the critical need for this current study which uses a combination of objective SB measurement and EMA.

#### Aim 2

It is expected that a portion of the CMD risk associated with CS-SB will be mediated by co-occurring lifestyle behaviors. For example, if a strong association is observed between TV viewing and CMD risk (in line with Aim 1), unhealthy food and alcohol consumption, which often occurs with TV viewing, may help explain this relationship^67,73,74^. Nevertheless, the overall hypothesis for Aim 2 is that CS-SB will continue to significantly associate with CMD risk following adjustment for clustered lifestyle behaviors.

#### Aim 3

We anticipate a range of intrapersonal, interpersonal, and physical environmental SEM factors will be associated with T-SB and CS-SB. Specifically, for intrapersonal factors, we hypothesize that individuals who report greater stress and burnout will be more sedentary^75^. In terms of interpersonal factors, we anticipate those who report poor social connectedness with family, friends, and their university community will engage with higher volumes of sedentary behavior^75^. Finally, for the physical environment level, we hypothesize those who are less aware of their engagement with SB and exist in dwellings that are more conducive to sedentary lifestyles will be more sedentary^76,77^.

## 4. Conclusions

CMD is a significant and worsening threat to public health in CBYA^14,18^. Sedentary behavior is strongly associated with increased CMD risk in CBYA^73,78^ and therefore there is a critical need for effective interventions to reduce SB in this population. This longitudinal study will provide novel insights into the association between CS-SB and CMD, examine whether the clustering of lifestyle behaviors that co-exist with CS-SB drives this association, and identify the socioecological predictors of CS-SB. Findings will support the development of an evidence-based, multi-level intervention to target SB reduction and mitigate CMD risk in CBYA. It will also provide a foundation for moving beyond current vague public health messaging like “sit less, move more”.

## Figures and Tables

**Table 1:** Outcomes (N = 14) that form the composite cardiometabolic disease risk score. The composite outcome will be comprised of the stated 14 novel and traditional outcomes of cardiometabolic health.

Method	Outcome	Unit	Full Name
**VICORDER**	cfPWV	m/s	Carotid-femoral PWV
	bfPWV	m/s	Brachial-femoral PWV
	cSBP	mmHg	Central systolic blood pressure
	MAP	mmHg	Mean arterial blood pressure
	AIx	%	Augmentation Index
**HRV**	RMSSD	*unitless*	Root Mean Square of Successive Differences between normal heartbeats
**Body composition**	Body Fat	%	Body fat percentage
	Trunk Body Fat	%	Trunk fat percentage
**Metabolic**	HDL-cholesterol	mmol/L	High-density lipoprotein cholesterol
	LDL-cholesterol	mmol/L	Low-density lipoprotein cholesterol
	Triglycerides	mmol/L	
	Fasting blood glucose	mmol/L	
	Insulin	mmol/L	
**Inflammatory**	C-reactive proteir	mg/L	

Abbreviations: AIx, Augmentation index; bfPWV, brachial-femoral pulse wave velocity; cSBP, Central systolic blood pressure; cfPWV, carotid-femoral pulse wave velocity; HDL, high-density lipoprotein; HRV, Heart rate variability; L, liter; LDL, low-density lipoprotein; m, meter; mg, milligram; mmol, millimole; MAP, Mean arterial blood pressure; mg, milligram; min, minute; mmHg, millimeters of mercury; RMSSD, Root mean square of successive differences; s, seconds

**Table 2:** Lifestyle behavior variables (n = 11) for Aim 2 of the CONTEXT-SB study. Lifestyle behaviors may potentially explain the relationship between context-specific SB and/or total-SB and CMD risk.

Lifestyle Category	Lifestyle Behavior	Method
**Physical Activity**	Light Physical Activity	activPAL
	Moderate-to-Vigorous Physical Activity	
**Diet**	Processed Food	DHQIII
	Breakfast	
	Fruits/Vegetables	
**Sleep**	Duration	SSM
	Quality	SSM
	Chronotype	RMEQ
**Substance Use**	Tobacco	WHO ASSIST
	Alcohol	
	Global	

Abbreviations: DHQIII, Diet Health Questionnaire III; RMEQ, Reduced Morning-Eveningness Questionnaire; SSM, SleepScore Max; WHO ASSIST, World Health Organization Alcohol, Smoking, and Substance Involvement Screening Test.

**Table 3:** Socioecological model variables (n = 16) included for Aim 3 of the CONTEXT-SB study. Socioecological model (SEM) predictors for our sedentary behavior variables. Cronbach’s α are derived from previous literature. The same 4 variables for the physical environment factor are presented for the college-environment and home-environment.

Questionnaire	Full Name	# Items	Variable	Cronbach α
**Intrapersonal (6 variables)**				
**PASE**	Physical Activity Self-Efficacy scale	5	Self-Efficacy	0.82^48^
**CBPAQ**	Cognitive Behavioral Physical Activity Questionnaire	5	Self-Regulation	0.78–0.89^49^
		5	Outcome Expectations	
		5	Personal Barriers	
**PSS-10**	Perceived Stress Scale	10	Psychological Stress	0.82^50^
**IBF-4**	Personal Burnout Scale	4	Personal Burnout	0.91^51^
**Interpersonal (2 variables)**				
**Social Norms and Social Support Scale**		3	Social Norms	0.74^52–53^
		3	Social Support	
**Physical environment (4 variables)**				
**PENF**	Physical Environmental Neighborhood Factors	5	Functionality	0.45–0.77^54^
		5	Safety	
		5	Aesthetics	
		5	Destination	

## Data Availability

Considering the scope and nature of this Study Protocol paper, no data were analyzed are directly pertinent to this manuscript.

## Abbreviations

BM: Bone mass
CBYA: College-based young adults
CMD: Cardiometabolic disease(s)
CML: Cardiometabolic Laborator
CONTEXT-SB: Cardiometabolic Outcome Negation Trough Early-adulthood ConteXT-specific Sedentary Behavior reduction
CS-SB: Context-specific sedentary behavior
DXA: Dual-Energy X-Ray Absorptiometry
EDTA: Ethylenediamine tetra-acetic acid
EKG: Electrocardiography
EMA: Ecological momentary assessment
FBG: Fasting blood glucose
FFM: Fat-free mass
FM: Fat mass
h: Hour
HbA1c: Glycated hemoglobin
HRV: Heart rate variability
Hz: Hertz
ICC: Intra-class correlation
ICG: Impedance cardiography
IRB: Institutional Review Board
LPA: Light physical activity
LPP: Longitudinal parallel-process
MET: Metabolic equivalent
MVPA: Moderate-to-vigorous physical activity
NIH: National Institute of Health
PWA: Pulse wave analysis
PWV: Pulse wave velocity
RMSSD: Root mean square of the successive difference
SB: Sedentary Behaviors
SEM: Socioecological model
T-SB: Total sedentary behavior
TV: Television
UNC: University of North Carolina at Chapel Hill
